# The impact of cardiovascular risk factors on cardiac structure and function: Insights from the UK Biobank imaging enhancement study

**DOI:** 10.1371/journal.pone.0185114

**Published:** 2017-10-03

**Authors:** Steffen E. Petersen, Mihir M. Sanghvi, Nay Aung, Jackie A. Cooper, José Miguel Paiva, Filip Zemrak, Kenneth Fung, Elena Lukaschuk, Aaron M. Lee, Valentina Carapella, Young Jin Kim, Stefan K. Piechnik, Stefan Neubauer

**Affiliations:** 1 William Harvey Research Institute, NIHR Biomedical Research Centre at Barts, Queen Mary University of London, Charterhouse Square, London, United Kingdom; 2 Division of Cardiovascular Medicine, Radcliffe Department of Medicine, University of Oxford, Oxford, United Kingdom; 3 Department of Radiology, Severance Hospital, Yonsei University College of Medicine, Seoul, South Korea; Kurume University School of Medicine, JAPAN

## Abstract

**Aims:**

The UK Biobank is a large-scale population-based study utilising cardiovascular magnetic resonance (CMR) to generate measurements of atrial and ventricular structure and function. This study aimed to quantify the association between modifiable cardiovascular risk factors and cardiac morphology and function in individuals without known cardiovascular disease.

**Methods:**

Age, sex, ethnicity (non-modifiable) and systolic blood pressure, diastolic blood pressure, smoking status, exercise, body mass index (BMI), high cholesterol, diabetes, alcohol intake (modifiable) were considered important cardiovascular risk factors. Multivariable regression models were built to ascertain the association of risk factors on left ventricular (LV), right ventricular (RV), left atrial (LA) and right atrial (RA) CMR parameters.

**Results:**

4,651 participants were included in the analysis. All modifiable risk factors had significant effects on differing atrial and ventricular parameters. BMI was the modifiable risk factor most consistently associated with subclinical changes to CMR parameters, particularly in relation to higher LV mass (+8.3% per SD [4.3 kg/m2], 95% CI: 7.6 to 8.9%), LV (EDV: +4.8% per SD, 95% CI: 4.2 to 5.4%); ESV: +4.4% per SD, 95% CI: 3.5 to 5.3%), RV (EDV: +5.3% per SD, 95% CI: 4.7 to 5.9%; ESV: +5.4% per SD, 95% CI: 4.5 to 6.4%) and LA maximal (+8.6% per SD, 95% CI: 7.4 to 9.7%) volumes. Increases in SBP were associated with higher LV mass (+6.8% per SD, 95% CI: 5.9 to 7.7%), LV (EDV: +4.5% per SD, 95% CI: 3.6 to 5.4%; ESV: +2.0% per SD, 95% CI: 0.8 to 3.3%) volumes. The presence of diabetes or high cholesterol resulted in smaller volumes and lower ejection fractions.

**Conclusions:**

Modifiable risk factors are associated with subclinical alterations in structure and function in all four cardiac chambers. BMI and systolic blood pressure are the most important modifiable risk factors affecting CMR parameters known to be linked to adverse outcomes.

## Introduction

Cardiovascular disease (CVD) accounts for the greatest burden of morbidity and mortality worldwide, resulting in 30% of all deaths annually. Coronary heart disease accounts for the greatest proportion of CVD [1].

Over the last sixty years, major organised efforts have been made to further understand the epidemiology of coronary heart disease, with the Framingham Heart Study [2], the MONICA project [3] and the INTERHEART study [4] amongst the most notable. These projects, along with many others [5–11], have established hypertension, cigarette smoking, diabetes, physical inactivity, obesity and elevated cholesterol levels to be the most important risk factors in the prediction and modification of CVD [12].

The UK Biobank project is a recent, large-scale, population-based prospective study. Alongside extensive collection of health questionnaire data, biological samples and physical measurements, it has utilised cardiovascular magnetic resonance (CMR)–the gold standard in generating accurate and reproducible measurements of cardiac morphology and function—to produce imaging-derived phenotypes [13]. This permits examination of the relationship between cardiovascular risk factors and changes in the structure and function of the cardiac chambers.

Previous studies investigating the association between cardiovascular risk factors and CMR measurements include only small numbers of subjects, do not examine all cardiac chambers or have used imaging techniques which are less precise and are typically no longer used in clinical practice (gradient recalled echo sequences) [14–16]. This study aims to examine and quantify the association between clinical and lifestyle factors and atrial and ventricular structure and function, changes in which are markers of subclinical cardiovascular disease.

## Methods

### Study population

The UK Biobank is a versatile scientific resource which collected questionnaire data, physical measurements and biological samples from 500,000 individuals in the UK [17]. Additionally, the UK Biobank imaging enhancement study is ongoing with the aim of performing, in a single visit, brain, heart, whole body, carotid artery, bone and joint imaging in 100,000 of the original 500,000 participants. CMR was selected as the modality of choice for heart imaging [13]. The study population presented here consists of the 5,065 individuals who underwent CMR examination as part of the pilot phase (April 2014 –August 2015) of the UK Biobank imaging enhancement. This study was covered by the general ethical approval for UK Biobank studies from the NHS National Research Ethics Service on 17th June 2011 (Ref 11/NW/0382). None of the authors had direct contact with the study participants.

#### Exclusion criteria

Participants reporting myocardial infarction, angina, heart failure, arrhythmias (including atrial fibrillation), cardiomyopathy, stroke or peripheral vascular disease were excluded from the analysis.

### CMR protocol and image analysis

The UK Biobank CMR protocol has been described in detail elsewhere [18]. Briefly, all examinations were performed on a wide-bore 1.5 Tesla scanner (MAGNETOM Aera, Syngo Platform VD13A, Siemens Healthcare, Erlangen, Germany). For cardiac function, long-axis cines and a complete short-axis stack of balanced steady-state free precession (bSSFP) cines, covering the left and right ventricle were acquired.

Typical parameters were as follows: TR/TE = 2.6/1.1ms, flip angle 80°, Grappa factor 2, voxel size 1.8 mm x 1.8 mm x 8 mm (6 mm for long axis). The actual temporal resolution of 32 ms was interpolated to 50 phases per cardiac cycle (~20 ms). No signal or image filtering was applied besides distortion correction.

Analysis of the left ventricle (LV), right ventricle (RV), left atrium (LA) and right atrium (RA) for all CMR examinations were performed manually by observers across two core laboratories according to pre-approved standard operating procedures using cvi^42^ post-processing software (Version 5.1.1, Circle Cardiovascular Imaging Inc., Calgary, Canada). LV papillary muscles were included in blood pool volumes and excluded from LV mass. Detailed descriptions of analysis methodology, including exemplar contours and intra- and inter-observer variability, have been previously described [19].

### Cardiovascular risk factor definitions

The following were considered to be important cardiovascular risk factors (12): age, sex (46% male), ethnicity (non-modifiable) and systolic blood pressure, diastolic blood pressure, current smokers (4%), levels of physical activity/exercise, body mass index (BMI), high cholesterol (12%), diabetes (4%) and alcohol intake (45%) (modifiable).

Ethnicity was categorised into a binary variable: Caucasian vs. non-Caucasian as non-Caucasians totalled only 4% of the total cohort. Systolic and diastolic blood pressures were defined as the mean of two measurements taken when participants attended their CMR examination using an automated monitor (Omron 705, OMRON Healthcare Europe, Hoofddorp, Netherlands). Smoking status was defined as a binary variable: current vs non-smokers at the time of CMR examination. Participants’ level of physical activity was determined by assessing frequency (number of days/week) and duration (minutes/day) of walking, moderate intensity and vigorous intensity exercise. A continuous value for the amount of physical activity—measured in metabolic equivalent (MET) minutes/week—was calculated by weighting different types of activity (walking, moderate or vigorous) by its energy requirements using values derived from the International Physical Activity Questionnaire (IPAQ) study [20]. Only participants <70 years of age had MET minutes/week calculated as the IPAQ is validated only in those between 15–69 years of age. Participants were deemed to have high cholesterol if they self-reported a diagnosis of “high cholesterol” or indicated use of “medication for cholesterol”. Diabetes status was determined by participants’ response to the binary questionnaire item “diabetes diagnosed by a doctor”. Participants indicating that they consumed alcohol on three or more occasions per week were defined as regular alcohol users.

### Data analysis and statistics

Results in Table 1 are presented as mean ± standard deviation (SD) unless stated otherwise. The directly measured volumes were skewed and therefore log_e_ transformation was used to normalise the distributions before analysis. Derived measurements such as ejection fraction were normally distributed and were not transformed. For each cardiac variable, outliers were defined as measurements more than three interquartile ranges below the first quartile or above the third quartile and removed from analysis. A separate multivariable linear regression model was fitted for each dependent variable. Dependent variables were LV mass, LV and RV end-diastolic volume, end-systolic volume, stroke volume, ejection fraction, and LA and RA maximal volume, minimal volume, stroke volume, ejection fraction. Independent variables were selected a priori and the same independent variables were included in the models for each outcome. Initial models were fitted with non-modifiable risk factors (age, sex, ethnicity and height) as the independent variables. Height was included to adjust for body size instead of body surface area in order to prevent attenuation of associations with BMI. Full models were then fitted with the addition of all the modifiable risk factors and results from the full models are presented in Tables 2–5. The coefficients from these models are adjusted for all other variables in the model. Model assumptions were checked using residual plots.

**Table 1:** Baseline characteristics of study cohort (n = 4,651).

	Women (n = 2518)	Men (n = 2133)
**Age**	61 ± 7.5	62 ± 7.5
**Non-Caucasian (%)**	2.7	3.7
**Height (cm)**	163 ± 6	176 ± 7
**BMI (kg/m2)**	26.2 ± 4.6	27.1 ± 3.9
**Systolic blood pressure (mmHg)**	133 ± 19	141 ± 16
**Diastolic blood pressure (mmHg)**	77 ± 10	81 ± 10
**Current smoker (%)**	3.5	5.3
**Regular alcohol use (%)**	37.8	52.6
**Exercise (MET minutes/week)**	2753 ± 2696	2910 ± 3056
**Raised choleseterol (%)**	14.1	9.8
**Diabetic (%)**	3.6	5.5

Values are expressed as mean ± standard deviation or as percentage of population. BMI = body mass index; MET = metabolic equivalent

**Table 2:** Multivariable regression models for LV parameters.

			LV mass			LV mass:volume ratio			LV end-diastolic volume			LV end-systolic volume			LV stroke volume			LV ejection fraction		
	R^2^																			
	Variability explained by full model (%)		65.5			21			52.2			41.6			41.2			6.7		
	Variability explained by modifiable risk factors (%)		27.4			11.3			11.2			4.1			12			1.4		
	Variable	Comparison	% change	LCI	UCI	% change	LCI	UCI	% change	LCI	UCI	% change	LCI	UCI	% change	LCI	UCI	Absolute change (%)	LCI	UCI
*Non-modifiable*	**Age**	Per 10 years	-2.9*	-3.8	-2	2.9*	1.9	3.9	-5.6*	-6.4	-4.7	-5.7*	-7	-4.5	-5.6*	-6.6	-4.7	0.1	-0.3	0.4
	**Sex**	F:M	-17.2*	-18.6	-15.8	-8.9*	-10.5	-7.3	-9.1*	-10.6	-7.6	-13.1*	-15.2	-11	-6.3*	-7.9	-4.6	1.9*	1.3	2.5
	**Ethnicity**	Non-Caucasian: Caucasian	-1.7	-4.8	1.5	4.0†	0.6	7.5	-6.4*	-9.3	-3.5	-8.0*	-12.1	-3.7	-5.1*	-8.3	-1.8	0.6	-0.5	1.8
	**Height**	Per SD (9.3 cm)	10.3*	9.4	11.2	-0.8	-1.6	0.1	11.1*	10.2	12	12.8*	11.5	14.1	9.9*	9	10.9	-0.6*	-0.9	-0.3
*Modifiable*	**BMI**	Per SD (4.3 kg/m^2^)	8.3*	7.6	8.9	3.3*	2.6	3.9	4.8*	4.2	5.4	4.4*	3.5	5.3	5.0*	4.3	5.7	0.1	-0.1	0.4
	**SBP**	Per SD (18.1 mmHg)	6.8*	5.9	7.7	2.2*	1.3	3.1	4.5*	3.6	5.4	2.0*	0.8	3.3	6.3*	5.3	7.2	0.9*	0.6	1.2
	**DBP**	Per SD (10.0 mmHg)	-1.9*	-2.7	-1.1	2.3*	1.4	3.1	-4.0*	-4.8	-3.2	-2.2*	-3.4	-1.1	-5.4*	-6.2	-4.5	-0.7*	-1	-0.4
	**Smoking**	Current vs. non	3.5†	0.8	6.2	5.0*	2.2	7.9	-1.4	-3.9	1.1	-0.2	-3.9	3.6	-2.4*	-5.1	0.4	-0.6	-1.5	0.4
	**Regular alcohol use**	>3 units/week vs. <3 units	1.4†	0.3	2.6	-0.4	-1.6	0.8	1.9*	0.8	3	1.3	-0.3	3	2.3*	1.1	3.6	0.2	-0.2	0.6
	**Exercise**	Per SD (2873 MET min/wk)	2.0*	1.4	2.5	-0.1	-0.7	0.5	2.1*	1.5	2.6	2.3*	1.5	3.2	1.9*	1.3	2.5	-0.1	-0.3	0.1
	**High cholesterol**	Yes:No	-1.4	-3.2	0.5	2.4†	0.4	4.4	-3.6*	-5.4	-1.8	-4.2*	-6.8	-1.6	-3.4*	-5.3	-1.4	0.1	-0.6	0.8
	**Diabetes**	Yes:No	-1.2	-4	1.7	3.3†	0.3	6.5	-4.3*	-7	-1.6	0.1	-3.9	4.4	-7.9*	-10.7	-4.9	-1.8*	-2.8	-0.8

* denotes significance level of <0.01,

^†^ denotes significance level of <0.05.

Coefficients presented have been adjusted for all other variables in the model. BMI = body mass index; DBP = diastolic blood pressure; F = female; LCI = lower confidence interval; LV = left ventricular; M = male; MET = metabolic equivalent; SD = standard deviation; SBP = systolic blood pressure; UCI = upper confidence interval.

**Table 3:** Multivariable regression models for RV parameters.

			RV end-diastolic volume			RV end-systolic volume			RV stroke volume			RV ejection fraction		
	R^2^													
	Variability explained by full model (%)		54.3			45.8			42.7			13.6		
	Variability explained by modifiable risk factors (%)		10.4			5			12.4			3.7		
	Variable	Comparison	% change	LCI	UCI	% change	LCI	UCI	% change	LCI	UCI	Absolute change (%)	LCI	UCI
*Non-modifiable*	**Age**	Per 10 years	-5.4*	-6.3	-4.5	-5.8*	-7.2	-4.5	-5.2*	-6.1	-4.2	0.2	-0.2	0.5
	**Sex**	F:M	-13.4*	-14.9	-12	-20.2*	-22.2	-18.2	-7.8*	-9.4	-6.1	0.5*	2.9	4.1
	**Ethnicity**	Non-Caucasian: Caucasian	-6.0*	-9	-2.9	-6.8*	-11.1	-2.2	-5.6*	-8.8	-2.4	0.3	-0.9	1.5
	**Height**	Per SD (9.3 cm)	10.6*	9.7	11.5	12.0*	10.6	13.4	9.5*	8.5	10.4	-0.5*	-0.9	-0.2
*Modifiable*	**BMI**	Per SD (4.3 kg/m2)	5.3*	4.7	5.9	5.4*	4.5	6.4	5.2*	4.6	5.9	-0.1	-0.3	0.2
	**SBP**	Per SD (18.1 mmHg)	2.8*	1.9	3.7	-1.4†	-2.7	-0.1	6.0*	5	6.9	1.7*	1.4	2.1
	**DBP**	Per SD (10.0 mmHg)	-3.3*	-4.1	-2.5	-1.1	-2.3	0.2	-4.9*	-5.8	-4.1	-1.0*	-1.3	-0.7
	**Smoking**	Current vs. non	-2.2	-4.8	0.5	-1.6	-5.4	2.4	-3.0†	-5.7	-0.2	-0.3	-1.3	0.6
	**Regular alcohol use**	>3 units/week vs. <3 units	1.1	0	2.3	-0.2	-1.9	1.5	2.2*	1	3.4	0.6*	0.1	1
	**Exercise**	Per SD (2873 MET min/wk)	2.2*	1.6	2.8	2.6*	1.8	3.5	1.9*	1.3	2.5	-0.2	-0.4	0
	**High cholesterol**	Yes:No	-4.5*	-6.3	-2.6	-5.7*	-8.3	-3	-4.0*	-5.9	-2	0.4	-0.3	1.1
	**Diabetes**	Yes:No	-5.3*	-8	-2.5	-3.2	-7.3	1.1	-7.1*	-9.9	-4.2	-1	-2.1	0.1

* denotes significance level of <0.01,

^†^ denotes significance level of <0.05.

Coefficients presented have been adjusted for all other variables in the model. BMI = body mass index; DBP = diastolic blood pressure; F = female; LCI = lower confidence interval; M = male; MET = metabolic equivalent; RV = right ventricular; SD = standard deviation; SBP = systolic blood pressure; UCI = upper confidence interval

**Table 4:** Multivariable regression models for LA parameters.

			LA maximal volume			LA minimal volume			LA stroke volume			LA ejection fraction		
	R^2^													
	Variability explained by full model (%)		18.3			13.8			17.2			4		
	Variability explained by modifiable risk factors (%)		10.6			7.7			9.7			1.6		
	Variable	Comparison	% change	LCI	UCI	% change	LCI	UCI	Absolute change (ml)	LCI	UCI	Absolute change (%)	LCI	UCI
*Non-modifiable*	Age	Per 10 years	-7.6*	-9.1	-6	-4.5*	-6.8	-2.3	-4.2*	-4.8	-3.6	-1.2*	-1.7	-0.7
	Sex	F:M	3.4*	0.4	6.4	4.7†	0.4	9.1	0.6	-0.4	1.7	-0.6	-1.4	0.3
	Ethnicity	Non-Caucasian: Caucasian	-0.4	-5.8	5.4	2.3	-5.5	10.7	-1	-3	1.1	-0.8	-2.4	0.7
	Height	Per SD (9.3 cm)	9.2*	7.7	10.8	12.7*	10.5	15	2.5*	2	3.1	-1.2*	-1.6	-0.8
*Modifiable*	BMI	Per SD (4.3 kg/m^2^)	8.6*	7.4	9.7	10.9*	9.3	12.5	2.6*	2.2	3	-0.8*	-1.1	-0.5
	SBP	Per SD (18.1 mmHg)	8.2*	6.6	9.9	8.8*	6.4	11.1	3.2*	2.7	3.8	-0.4	-0.8	0
	DBP	Per SD (10.0 mmHg)	-7.0*	-8.4	-5.7	-8.2*	-10.1	-6.3	-2.7*	-3.2	-2.1	0.7*	0.3	1.1
	Smoking	Current vs. non	-4.2	-8.5	0.3	-2.1	-8.3	4.5	-2.3*	-4	-0.6	-0.9	-2.2	0.3
	Regular alcohol use	>3 units/week vs. <3 units	2.4†	0.4	4.5	2.5	-0.4	5.4	1.0*	0.3	1.8	-0.1	-0.6	0.5
	Exercise	Per SD (2873 MET min/wk)	2.4*	1.4	3.4	2.3*	0.8	3.7	1.0*	0.6	1.3	0.1	-0.2	0.4
	High cholesterol	Yes:No	-2.5	-5.7	0.8	-2.2	-6.7	2.5	-1	-2.3	0.2	-0.4	-1.3	0.5
	Diabetes	Yes:No	-6.5*	-11.1	-1.8	-2.9	-9.6	4.2	-3.6*	-5.5	-1.8	-1.2	-2.6	0.1

* denotes significance level of <0.01,

^†^ denotes significance level of <0.05.

Coefficients presented have been adjusted for all other variables in the model. BMI = body mass index; DBP = diastolic blood pressure; F = female; LCI = lower confidence interval; M = male; MET = metabolic equivalent; RV = right ventricular; SD = standard deviation; SBP = systolic blood pressure; UCI = upper confidence interval.

**Table 5:** Multivariable regression models for RA parameters.

			RA maximal volume			RA minimal volume			RA stroke volume			RA ejection fraction		
	R^2^													
	Variability explained by full model (%)		27.3			30.8			9.2			8.9		
	Variability explained by modifiable risk factors (%)		3			2			2.3			0.9		
	Variable	Comparison	% change	LCI	UCI	% change	LCI	UCI	Absolute change (ml)	LCI	UCI	Absolute change (%)	LCI	UCI
*Non-modifiable*	*Age*	Per 10 years	0	-1.6	1.6	1.8	-0.1	3.7	-0.7	-1.4	0	-1.0*	-1.6	-0.5
	*Sex*	F:M	-10.0*	-12.5	-7.4	-15.7*	-18.5	-12.9	-0.9	-2.2	0.3	3.7*	2.7	4.7
	*Ethnicity*	Non-Caucasian: Caucasian	-5.8†	-10.7	-0.6	-5.6	-11.4	0.6	-2.2	-4.5	0.2	-0.1	-2.1	1.8
	*Height*	Per SD (9.3 cm)	12.3*	10.8	13.9	14.7*	12.8	16.6	2.9*	2.3	3.5	-1.2*	-1.7	-0.7
*Modifiable*	*BMI*	Per SD (4.3 kg/m2)	0.4	-0.6	1.4	1.7*	0.5	2.9	-0.6†	-1	-0.1	-0.7*	-1.1	-0.4
	*SBP*	Per SD (18.1 mmHg)	1.5†	0.1	3.1	1	-0.7	2.8	0.9†	0.2	1.5	0.4	-0.2	0.9
	*DBP*	Per SD (10.0 mmHg)	-3.1*	-4.5	-1.7	-3.5*	-5.1	-1.9	-1.0*	-1.7	-0.4	0.2	-0.4	0.7
	*Smoking*	Current vs. non	-7.4*	-11.4	-3.2	-6.5†	-11.2	-1.4	-2.9*	-4.8	-0.9	-0.6	-2.2	1
	*Regular alcohol use*	>3 units/week vs. <3 units	2.4*	0.5	4.4	3.0†	0.6	5.3	0.4	-0.5	1.3	-0.4	-1.1	0.3
	*Exercise*	Per SD (2873 MET min/wk)	2.3*	1.3	3.2	2.1*	1	3.3	0.7*	0.2	1.1	0.1	-0.3	0.4
	*High cholesterol*	Yes:No	-6.4*	-9.4	-3.4	-4.3†	-7.8	-0.7	-2.8*	-4.2	-1.4	-1.4†	-2.6	-0.2
	*Diabetes*	Yes:No	-7.9*	-12.4	-3.3	-8.1*	-13.3	-2.6	-2.8*	-4.9	-0.6	-0.1	-1.9	1.7

* denotes significance level of <0.01,

^†^ denotes significance level of <0.05.

Coefficients presented have been adjusted for all other variables in the model. BMI = body mass index; DBP = diastolic blood pressure; F = female; LCI = lower confidence interval; M = male; MET = metabolic equivalent; RV = right ventricular; SD = standard deviation; SBP = systolic blood pressure; UCI = upper confidence interval.

As the outcome variables within each region were correlated, a multivariate analysis was conducted for the full models and an overall test for the association of each predictor variable over the combined outcome variables was made using Pillai’s trace (S1 Table); mass:volume ratio was excluded from this analysis due to multicollinearity.

The coefficient of determination (R^2^) was obtained for each response variable to determine the proportion of variation explained by the independent variables. Partial R^2^ values were calculated as an indication of the proportion of variability explained by the addition of the modifiable factors to the model. R^2^ values for the full model and the partial R^2^ are presented in the tables.

Where cardiac variables had been log-transformed, the beta coefficients and confidence intervals were anti-logged and expressed as a percentage change. For continuous independent variables, results were presented as the change in the variable associated with an increase of one standard deviation (SD) of the independent variable with 95% confidence intervals (CI).

The three cardiovascular risk factors that contributed most to the R^2^ value for each CMR parameter were identified and the proportion of the contribution, expressed as a percentage, was calculated. Multicollinearity was assessed using the variance inflation factor.

Statistical analysis was performed using R (version 3.3.1) Statistical Software [21].

## Results

A total of 5,065 CMR examinations were analysed. 90 participants were excluded due to either CMR data being of insufficient quality or the CMR identifier did not match the participant identifier. A further 323 (6.5%) participants had known CVD and were excluded. The study population, therefore, consisted of 4,651 participants (2,133 [45.9%] males and 2,518 [54.1%] females).

**Fig 1:**
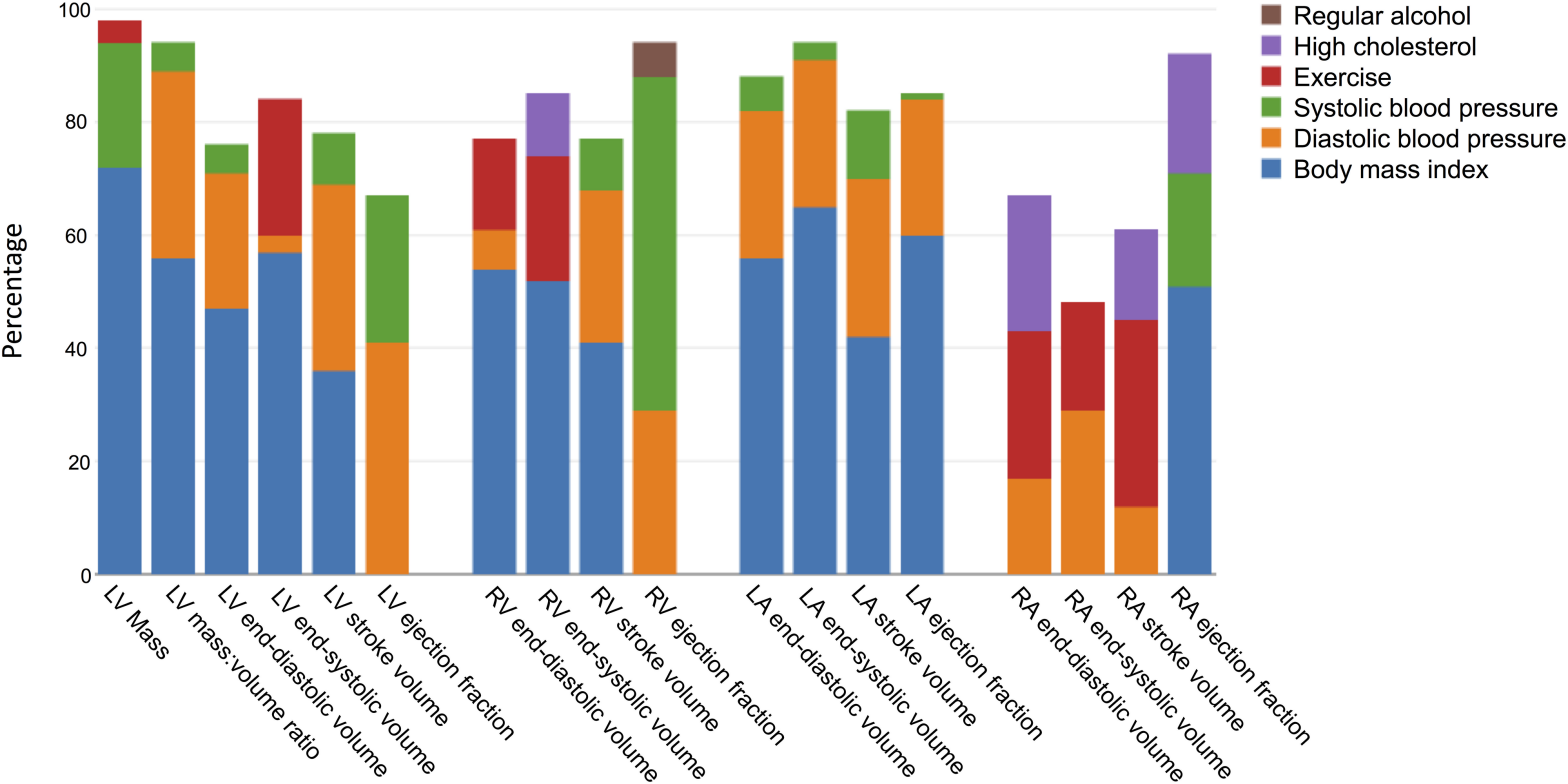
Modifiable risk factors and contribution to adjusted R^2^ as a percentage. Body mass index, systolic and diastolic blood pressure are the modifiable risk factors which most consistently contribute to alterations in cardiac structure and function.

Baseline characteristics for the study population, divided by sex, are presented in Table 1. Multiple linear regression models for LV, RV, LA and RA measurements are presented in Tables 2–5, respectively. Each table contains separate multivariable models and R^2^ values relating to the chamber-specific CMR variables. Non-modifiable risk factors explained a higher percentage of the overall variability (total R^2^) than modifiable risk factors. The three modifiable cardiovascular risk factors which contributed most to the proportion of variation—as determined by R^2^ value—are depicted in Fig 1.

### Left ventricle

Multiple regression models, R^2^ values and partial R^2^ values for LV parameters are presented in Table 2. Higher LV mass was associated with increasing BMI (+8.3% per SD [4.3 kg/m^2^], 95% confidence interval [CI]: 7.6 to 8.9%), systolic blood pressure (+6.8% per SD [18 mmHg], CI: 5.9 to 7.7%), current smoking (+3.5% vs non-smokers, CI: 0.8 to 6.2%), regular alcohol use (+1.4% vs <3 units/week, CI: 0.3 to 2.6%) and exercise (+2.0% per SD [2,873 MET minutes/week], CI: 1.4 to 2.5%). LV mass was lower with increasing diastolic blood pressure (-1.9% per SD [10 mmHg], CI: -2.7 to -1.1%). High cholesterol or diabetes did not significantly affect LV mass. Fig 1 demonstrates the majority of variability attributable to modifiable risk factors for changes in LV mass was explained by BMI (72%) and systolic blood pressure (22%). Changes in LV mass:volume ratio were most significantly associated with increases in BMI (+3.3% per SD, CI: 2.6 to 3.9%), diastolic blood pressure (+2.3% per SD, CI: 1.4 to 3.1%) and systolic blood pressure (+2.2% per SD, CI: 1.3 to 3.1%). Unlike LV mass, a higher LV mass:volume ratio was demonstrated for both increases in systolic and diastolic blood pressure.

LV end-diastolic volume and end-systolic volume were positively associated with BMI (EDV: +4.8% per SD, CI: 4.2 to 5.4%; ESV: +4.4% per SD, CI: 3.5 to 5.3%), systolic blood pressure (EDV: +4.8% per SD, CI: 4.2 to 5.4%; ESV: +4.4% per SD, CI: 3.5 to 5.3%), and exercise (EDV: +2.1% per SD, CI: 1.5 to 2.6%; ESV: +2.3% per SD, CI: 1.5 to 3.2%). A lower LV end-diastolic volume was observed with higher diastolic blood pressures (-4.0% per SD, CI: -4.8 to -3.2%) and participants with high cholesterol (-3.6%, CI: -5.4 to -1.8%) and diabetes (-4.3%, CI: -7.0 to -1.6%). For LV end-systolic volume, these associations were only seen for diastolic blood pressure (-2.2% per SD, CI: -3.4 to -1.1%) and high cholesterol (-4.2%, CI: -6.8 to -1.6%). For LV end-diastolic volume, BMI (47%) and diastolic blood pressure (24%) contributed most to the explanation of variability, but note that their effect sizes were in opposite directions. For LV end-systolic volume, BMI (57%) and exercise (24%) were important contributors, both of which were associated with higher LV end-systolic volume.

The impact of cardiovascular risk factors on LV stroke volume was similar to that demonstrated for LV end-diastolic volume. Increases in BMI (+5.0% per SD, CI: 4.3 to 5.7%), systolic blood pressure (+6.3% per SD, CI: 5.3 to 7.2%), regular alcohol use (+2.3%, CI: 1.1 to 3.6%) and exercise (+1.9% per SD, CI: 1.3 to 2.5%) resulted in higher LV stroke volume. Conversely, increases in diastolic blood pressure (-5.4% per SD, CI: -6.2 to -4.5%) and presence of high cholesterol (-3.4%, CI: -5.3 to -1.4%) or diabetes resulted in lower LV stroke volume. Notably, diabetic participants demonstrated an 8% reduction in LV stroke volume (CI: -10.7 to -4.9%) compared to participants without diabetes in the multivariable model.

Three cardiovascular risk factors were significantly associated with changes in LV ejection fraction. An increase in systolic blood pressure by one SD (18 mmHg) was associated with an absolute change of +0.9% in LV ejection fraction (CI: 0.6 to 1.2%). Increases in diastolic blood pressure (one SD = 10 mmHg) and presence of diabetes were associated with a 0.7% (CI: -1.0 to -0.4%) and 1.8% (CI: -2.8 to -0.8%) reduction in LV ejection fraction, respectively.

### Right ventricle

Multivariable models for RV CMR parameters are presented in Table 3. Higher RV end-diastolic volume was associated with increases in BMI (+5.3% per SD, CI: 4.7 to 5.9%), systolic blood pressure (+2.8% per SD, CI: 1.9 to 3.7%) and exercise (+2.2% per SD, CI: 1.6 to 2.8%). RV end-diastolic volume demonstrated an inverse relationship with increases in diastolic blood pressure (-3.3% per SD, CI: -4.1 to -2.5%), high cholesterol (-4.5%, CI: -6.3 to -2.6%) and presence of diabetes (-5.3%, CI: -8.0 to -2.5%). For RV end-systolic volume, there were positive associations with BMI (+5.4% per SD, CI: 4.5 to 6.4%) and exercise (+2.6% per SD, CI: 1.8 to 3.5%) and negative associations with increases in systolic blood pressure (-1.4% per SD, CI: -2.7 to -0.1%) and high cholesterol (-5.7% per SD, CI: -8.3 to -3.0%). In contrast to the LV, where blood pressure played a significant contributory role in explanation of the variance in LV end-diastolic and LV end-systolic volume measurements, for the equivalent RV measurements, the variance was attributable to increases in BMI and exercise.

All modifiable risk factors had significant impact on RV stroke volume. RV stroke volume was higher with increases in BMI (+5.2% per SD, CI: 4.6 to 5.9%), systolic blood pressure (+6.0% per SD, CI: 5.0 to 6.9%), regular alcohol use (+2.2%, CI: 1.8 to 3.5%) and physical activity (+1.9% per SD, CI: 1.3 to 2.5%). It was lower with increases in diastolic blood pressure (-4.9% per SD, CI: -5.8 to -4.1%), being a current smoker (-3.0%, CI: -5.7 to -0.2%), high cholesterol (-4.0%, CI: -5.9 to -2.0%) or presence of diabetes (-7.1%, CI: -9.9 to -4.2%). RV ejection fraction was higher with increasing systolic blood pressure (+1.7% per SD, CI: 1.4 to 2.1%) and regular alcohol use (0.6%, CI: 0.1 to 1.0%); it was lower with increasing diastolic blood pressure (-1.0% per SD, CI: -1.3 to -0.7%). Variance in RV ejection fraction was primarily explained by blood pressure.

### Atria

LA maximal volume was larger with increasing BMI (+8.6% per SD, CI: 7.4 to 9.7%), systolic blood pressure (+8.2% per SD, CI: 6.6 to 9.9%), regular alcohol use (+2.4%, CI: 0.4 to 4.5%) and exercise (+2.4% per SD, CI: 1.4 to 3.4%) (Table 4). It was smaller with increasing diastolic blood pressure (-7.0% per SD, CI: -8.4 to -5.7%) and presence of diabetes (-6.5%, CI: -11.1 to -1.8%). BMI, systolic and diastolic blood pressures were demonstrated to have the greatest impact on all LA parameters. RA maximal volume was smaller with increasing diastolic blood pressure (-3.1% per SD, CI: -4.5 to -1.7%), high cholesterol (-6.4%, CI: -9.4 to -3.4%), presence of diabetes (-7.9%, CI: -12.4 to -3.3%) and being a current smoker (-7.4%, CI: -11.4 to -3.2%) (Table 5). It was larger with exercise (-2.4% per SD, CI: -0.5 to -4.4%) and regular alcohol use (-2.4%, CI: -0.5 to -4.4%). The RA ejection fraction was negatively associated with increasing BMI (-0.7% per SD, CI: -1.1 to -0.4%) and presence of high cholesterol (-1.4%, CI: -2.6 to -0.2%).

## Discussion

The present study demonstrated that the presence of modifiable cardiovascular risk factors explained a meaningful proportion of the variance observed in LV mass and LV, RV, LA and RA volumes, as measured by CMR in a cohort of 4,651 participants without known cardiovascular disease.

In multivariable regression models including adjustment for non-modifiable risk factors (age, sex, ethnicity) and height, all modifiable risk factors (BMI, systolic blood pressure, diastolic blood pressure, current smoking, regular alcohol intake, physical activity/exercise level, high cholesterol and presence of diabetes) had significant effects on various atrial and ventricular CMR parameters. BMI was the modifiable cardiovascular risk factor most consistently associated with subclinical changes to CMR parameters, particularly in relation to higher LV mass and LV, RV and LA volumes. Increases in systolic blood pressure were associated with greater LV and RV volumes whereas the inverse effect was observed with increases in diastolic blood pressure. Increasing levels of exercise resulted in higher LV, RV and LA volumes whereas the presence of diabetes, or high cholesterol generally resulted in smaller chamber volumes and ejection fractions.

The vast majority of large-scale studies investigating the determinants of CVD have, by design, focused upon clinical end-points—that is, development of specific disease states associated with clinical symptoms or diagnostic criteria. From these, the determinants of CVD (i.e. risk factors) have been ascertained. However, it is known that CVD, with its mostly atherosclerosis-driven pathophysiology, is generally a chronic process; plaque formation has been demonstrated to occur more than twenty years prior to the onset of cardiovascular symptoms [22]. It is logical, therefore, that patients with cardiovascular risk factors, but no clinical disease, might exhibit subclinical alterations in cardiac structure and function. This is subclinical cardiac remodelling and these changes have been demonstrated to impact upon cardiovascular morbidity and mortality: increased LV mass is a well-studied predictor of CVD [23–26]. Furthermore, LV end-systolic volume, LV end-diastolic volume, LV ejection fraction, RV end-diastolic volume, RV ejection fraction, LA and RA volumes have also been demonstrated to be of prognostic importance in the context of CVD [27–34].

CMR is the most accurate and reproducible cardiac imaging modality [35]; coupled with the large cohort size in this study, it has permitted detection of subclinical differences in mass, volumes and function in relation to cardiovascular risk factors. Whilst these subtle alterations may not result in any discernible impact to an individual at a single time point, if these differences persist they will eventually lead to clinically and prognostically relevant changes in cardiovascular outcomes. Furthermore, given that this study demonstrates a proportion of these alterations relate to modifiable risk factors, it is important to consider that, at a population level, these changes—and the question of how to tackle them—assume public health importance.

This study’s findings regarding associations of cardiovascular risk factors and LV and RV measurements are broadly in line with previous population studies utilising CMR [14–16] and echocardiography [36]. To our knowledge, no similar studies exist examining the left or right atrium and their relationship to modifiable risk factors. Our study points to increasing BMI and blood pressure to be of greatest importance to changes to atrial structure and function. These findings may be of relevance regarding the increasing prevalence of atrial fibrillation, and, from a public health perspective, hold clues to the prevention of this condition which has an incidence that is markedly increasing [37]. The INTERHEART study, which determined the health behaviours and disease states most associated with risk of myocardial infarction, identified cigarette smoking and abdominal obesity as the most important risk factors in a Western European population [4]. It is notable, however, that our study population had a far lower proportion of current smokers (4.4%) compared to the national average of the UK (19%) [38] or the USA (15%) [39], most likely because the UK Biobank cohort is, by definition, aged 40–80 years.

In our study, increasing BMI was established to be a major cause of variation observed in CMR measurements across cardiac chambers (Fig 1). The Pathobiological Determinants of Atherosclerosis in Youth (PDAY) study has demonstrated that obesity significantly increases the presence of fatty streaks and atherosclerotic plaques even in people of young age (<34 years) [40], and there is compelling evidence concerning remodelling of both the left and right ventricle in studies undertaken using CMR [41,42]. Systolic and diastolic blood pressure were also important contributors to the proportion of variability observed for many ventricular and atrial parameters. It is notable, however, that besides RA ejection fraction, systolic blood pressure invariably resulted in higher mass or volumes whilst diastolic blood pressure resulted in lower atrial and ventricular parameters for both the left and right heart. This observation has been highlighted by the Multi-Ethnic Study of Atherosclerosis and the Framingham study [43]. Smaller ventricular cavity volumes with increasing diastolic blood pressure might be explained by a decrease in chamber distensibility during diastole due to increased myocardial stiffness in the context of persistently high afterload resulting from hypertension.

Whilst only risk factors that have been demonstrated to play consistent roles in explanation of R^2^ values have been discussed here, what is clear from our results is that all modifiable risk factors examined in this study impact on functional parameters for all of the cardiac chambers to a greater or lesser extent. The INTERHEART study demonstrated that 92% of the risk of an initial myocardial infarction is accounted for by the modifiable risk factors [4]. Given that the CMR parameters described here are derived from measurements of cardiac structures, each of which has a physical attachment to two other chambers, it is evident that in models neither including adjoining chambers, nor other determinants of physical structure such as genes, a full explanation through multivariable regression models is not possible. It remains the case, however, that at the subclinical stage, modifiable risk factors affect cardiac structure and function and that many of these changes have been linked with adverse cardiovascular outcomes.

Limitations of this study include its cross-sectional nature and therefore the inability to assess the impact of risk factors on the cardiac chambers over time. Secondly, neither cholesterol nor glucose data was available for use in this study at this point, and instead data on these conditions was based on questionnaire responses provided by participants.

## Conclusions

In summary, in this large population-based cohort free of clinical cardiovascular disease, modifiable risk factors were associated with subclinical alterations of all four cardiac chambers. BMI, systolic and diastolic blood pressures are the most important modifiable cardiovascular risk factors affecting CMR parameters known to be associated with adverse outcomes.

It is hoped that these findings prove valuable for three reasons: firstly, by providing data as to how routinely assessed CMR parameters are affected by common cardiovascular risk factors, we provide useful information to clinicians seeking to ascertain whether measurements falling outside reference ranges are attributable to pathology or are in keeping with an individual’s risk factor profile. Secondly, in the research setting, we provide information of use to those wishing to design clinical trials or perform sample size calculations where CMR measurements are to be used as surrogate markers. Finally, at the population health level, we hope that illustrating the quantifiable impact of risk factors at the subclinical stage, particularly with regards to BMI and hypertension, might inform future public health strategies regarding modification of individuals’ risk.

## Supporting information

S1 Table Multivariate tests for each predictor variable from the full models. Data shown in this table is for overall tests of the association of each predictor variable over the combined outcome variables using Pillai’s trace.
(XLSX)

## Abbreviations

BMI: body mass index
bSSFP: balanced steady-state free precession
CMR: cardiovascular magnetic resonance
CVD: cardiovascular disease
LA: left atrium
LV: left ventricle
MET: metabolic equivalent
RA: right atrium
RV: right ventricle
SD: standard deviation
TE: echo time
TR: repetition time
